# Concurrent Nemolizumab-Induced Cutaneous Adverse Events and Asthma Exacerbation in a Patient With Prurigo Nodularis With Resolution of Both Conditions After Switching to Dupilumab

**DOI:** 10.7759/cureus.96605

**Published:** 2025-11-11

**Authors:** Yoshihito Mima, Masako Yamamoto, Ken Iozumi

**Affiliations:** 1 Department of Dermatology, Tokyo Metropolitan Police Hospital, Nakano City, JPN

**Keywords:** asthma, cutaneous adverse events, interleukin-31, nemolizumab, t helper 2

## Abstract

Prurigo nodularis (PN) is a chronic pruritic inflammatory disease driven by Th2-dominant immune dysregulation and neural hyperactivity. Conventional therapies often fail, while biologics such as dupilumab and nemolizumab have shown efficacy. However, nemolizumab can cause cutaneous adverse events (cAEs) and occasional asthma exacerbation. An 82-year-old woman with long-standing PN and well-controlled asthma developed cAEs and asthma exacerbation one month after initiating nemolizumab, an IL-31 receptor antagonist. Although nemolizumab markedly improved pruritus and PN lesions, acute eczematous eruptions and erythematous papules appeared on previously unaffected skin, accompanied by worsening asthma symptoms. After switching to dupilumab (IL-4/IL-13 inhibitor), both cAEs and asthma improved, with complete resolution within two months. The mechanism underlying nemolizumab-induced cAEs and asthma remains unclear, but activation of Th2-driven inflammation following IL-31 blockade has been proposed. To our knowledge, this is the first reported case of concurrent nemolizumab-induced cAEs and asthma exacerbation in a patient with PN, successfully managed with dupilumab. Given that asthma exacerbations have occurred only in patients with preexisting asthma, careful monitoring is warranted when administering nemolizumab. Further studies are needed to clarify its mechanism and management strategies.

## Introduction

Prurigo nodularis (PN) is a chronic inflammatory skin disorder characterized by intensely pruritic papules and nodules. Similar to atopic dermatitis (AD), PN is exacerbated by the “itch-scratch cycle,” with dysregulation of immune and neural pathways playing a central role in its pathogenesis. PN is also associated with several comorbidities, including eczema, psychiatric disorders, malignancies, hepatic and renal dysfunction, and diabetes mellitus [1]. In addition to T helper (Th)2-dominant chronic inflammation, aberrant proliferation of nerve fibers and excessive neuropeptide production contribute to its disease development [2]. Th2 cytokines, including IL-4, IL-13, and IL-31, drive a Th2-mediated inflammatory response that plays a central role in allergic pathophysiology [2]. Conventional treatments include topical corticosteroids, phototherapy, and systemic immunosuppressants such as cyclosporine, but in recent years, biologic therapies such as dupilumab and nemolizumab have emerged as promising alternatives [3-5]. Dupilumab, an interleukin (IL)-4/IL-13 inhibitor, has demonstrated significant improvement in both pruritus and cutaneous manifestations, leading to its approval for PN [3]. Similarly, nemolizumab, an IL-31 receptor antagonist, has shown statistically significant improvements in both itch and lesion severity compared with placebo and was approved for PN in 2024 [4,5]. However, approximately 10-30% of patients receiving nemolizumab experience cutaneous adverse events (cAEs), including edematous erythema or acute eczema, Malassezia folliculitis, or erythema multiforme [6-8]. Although the fundamental mechanisms underlying the development of cAEs remain unclear, recent observations suggest that transient, paradoxical activation of Th2 inflammation following IL-31 blockade may play a role. Standardized strategies for the prevention and management of cAEs have not yet been established, and caution is warranted, as severe or generalized cAEs can necessitate treatment discontinuation [6-8]. In addition to cAEs, nemolizumab has been reported to exacerbate asthma. In a 16-week phase 3 trial of nemolizumab for PN, asthma exacerbation occurred in 1.1% of patients, all of whom had a prior history of asthma, though all cases were mild-to-moderate in severity [4]. Herein, we report a case of a patient with PN who developed concurrent cAEs and asthma exacerbation following nemolizumab administration.

## Case presentation

**Figure 1:**
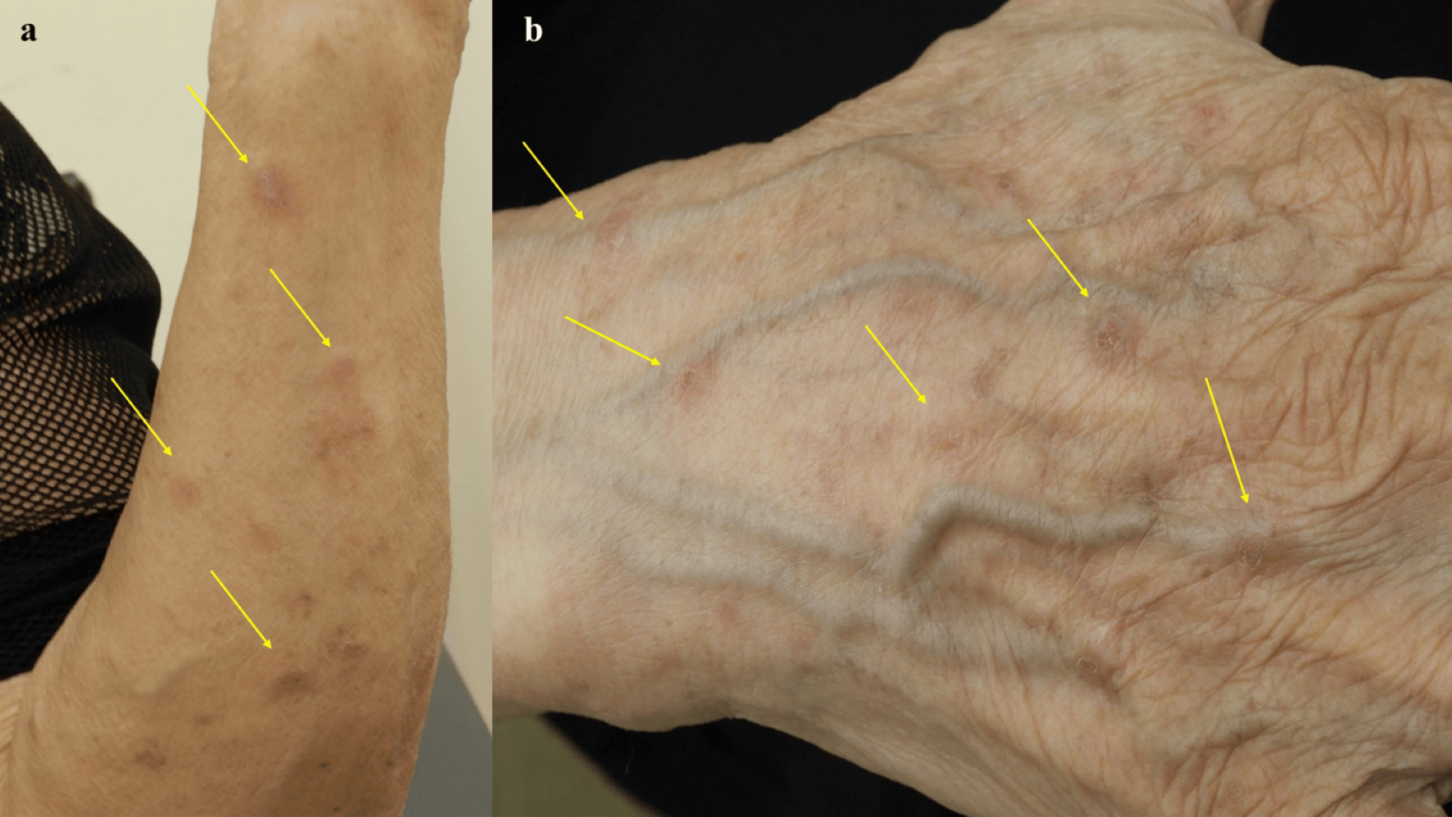
Approximately a month after receiving a 60 mg dose of nemolizumab, the patient developed multiple eczematous lesions on the left upper limb (a) and numerous erythematous papules on the right upper limb (b) (yellow arrows), while the subjective pruritus had almost completely resolved.

An 82-year-old woman presented with PN accompanied by severe pruritus. Her condition had been refractory to topical corticosteroids for over 20 years. Comorbid asthma had been well controlled for more than five years with an inhaled corticosteroid/long-acting β₂-agonist (ICS/LABA) combination. To achieve rapid symptom relief, nemolizumab (60 mg) was initiated. One month after the first dose, the Peak Pruritus Numerical Rating Scale (PP-NRS) score improved from 8 to 2, and the PN-Investigator’s Global Assessment (IGA) score decreased from 3 to 2, indicating a favorable clinical response. However, at the same time, acute eczematous erythema and erythematous papules without pruritus appeared on the upper extremities, in areas previously unaffected by PN (Figures 1).

**Figure 2:**
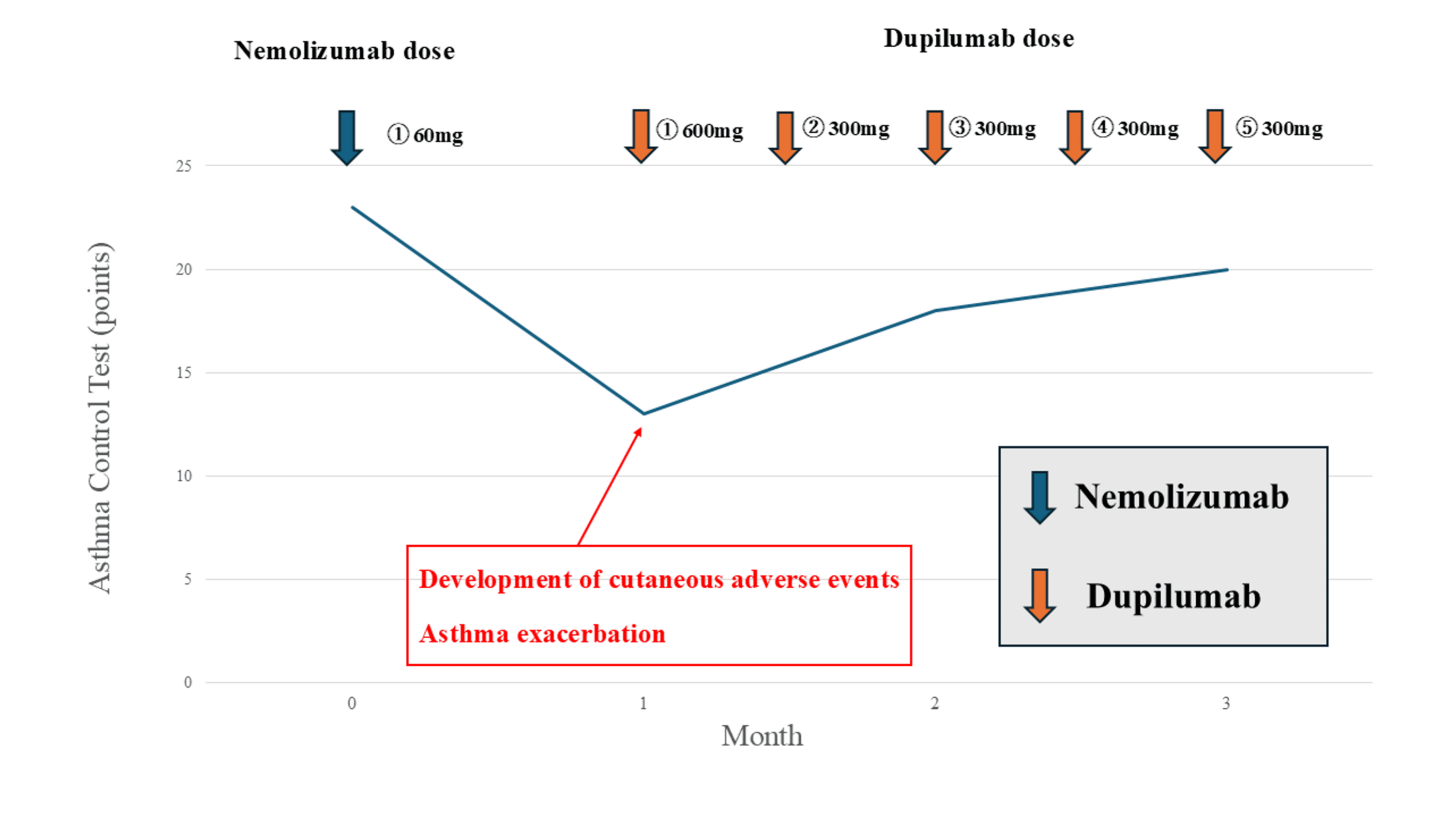
Course of drug administration and changes in the Asthma Control Test (ACT) score. Following the initial nemolizumab dose, the ACT score worsened from 23 to 13. After switching to dupilumab (600 mg loading dose, followed by 300 mg every two weeks), the ACT score improved to 18 after one month and to 20 after two months. Two months after switching, the eczematous eruptions and erythematous papules had shown marked regression.

In addition, her asthma symptoms worsened, requiring the use of a short-acting β₂-agonist (SABA) as rescue medication. She also developed nocturnal awakenings and insomnia, and her Asthma Control Test (ACT) score declined from 23 to 13 (Figure 2) [9].

Consequently, nemolizumab was discontinued and replaced with dupilumab (a 600 mg loading dose, followed by 300 mg every two weeks). After switching, both asthma symptoms and cAEs gradually improved. Two months later, her ACT score recovered to 20 (Figure 2), asthma was again well controlled with her previous ICS/LABA regimen alone, and the cutaneous adverse events had almost completely resolved under dupilumab treatment (Table 1).

**Table 1:** Longitudinal treatment course. Abbreviations: PP-NRS: Peak Pruritus–Numeric Rating Scale, PN-IGA: Prurigo Nodularis–Investigator’s Global Assessment, ACT: Asthma Control Test, cAEs: Cutaneous Adverse Events

Month	0	1	2	3
Treatment	Nemolizumab	Dupilumab	Dupilumab	Dupilumab
PP-NRS	8	2	1	0
PN-IGA	3	2	2	1
ACT	23	13	18	20
cAEs	✖	Onset	➡	Resolution

Given the absence of medication changes and the patient’s stable asthma control over the past several years, drug-related or seasonal factors were considered unlikely. We therefore deemed nemolizumab-induced asthma worsening to be the most plausible explanation and concluded that the drug may have contributed not only to the cutaneous adverse events but also to the exacerbation of asthma.

## Discussion

In this case, pruritus improved following nemolizumab administration; however, cAEs such as acute eczematous eruptions and erythematous papules, along with asthma exacerbation, developed concurrently.

The exact mechanisms underlying nemolizumab-induced cAEs remain unclear, but the activation of Th2-driven inflammation following IL-31 inhibition has been proposed as a contributing factor [10-12]. In IL-31 knockout mice, OSM (Oncostatin M)-dependent upregulation of IL-4 and IL-13 expression has been observed, supporting the hypothesis of enhanced Th2 inflammation [10,11]. Moreover, in clinical trials of nemolizumab, approximately 5% of patients exhibited elevated serum thymus and activation-regulated chemokine (TARC) levels - a Th2-specific chemokine - which were associated with the development of edematous erythema and acute eczema. Case reports have also suggested a correlation between the course of TARC elevation and the onset of cAEs [12-15]. Furthermore, although the mechanisms of nemolizumab-induced asthma exacerbation remain uncertain, nemolizumab has also been reported to induce Th2-driven inflammation, resulting in exacerbating asthma [16]. IL-4 and IL-13 are key upstream mediators of type 2 inflammation in asthma, and dupilumab, by blocking these cytokines, has been shown to significantly reduce the rate of severe exacerbations and rapidly improve asthma control [17].

In the present case, both cAEs and asthma exacerbation occurred simultaneously after nemolizumab administration, suggesting that Th2 inflammation activation induced by nemolizumab may represent a common underlying mechanism [10-17]. Switching to dupilumab, which suppresses Th2 inflammation, led to resolution of both the cutaneous and respiratory manifestations. Katsuta et al. also reported a case in which nemolizumab treatment led to worsening of AD and pre-existing asthma, further supporting our hypothesis that the activation of Th2 inflammation due to nemolizumab may contribute to the coexistence of both conditions [18]. To our knowledge, this is the first report describing concurrent nemolizumab-induced cAEs and asthma exacerbation in a patient with PN. In our case, the condition was successfully managed by switching to dupilumab, whereas Katsuta et al. managed their case by discontinuing nemolizumab and administering oral corticosteroids [18]. Currently, no established management strategy exists for asthma exacerbation induced by nemolizumab, and no clear algorithm defines when to discontinue the drug or how to appropriately escalate asthma treatment. Therefore, treatment decisions should be individualized based on symptom severity. Notably, in previous clinical trials, asthma exacerbations associated with nemolizumab occurred exclusively in patients with a prior history of asthma [4], underscoring the need for close monitoring when administering nemolizumab to such patients. This report has several limitations. This report is based on a single case with limited generalizability, and we were unable to assess Th2-related biomarkers, such as IgE, TARC, eosinophil counts, or serum IL-4/13 levels. Therefore, any discussion regarding the underlying Th2-driven mechanisms remains hypothetical. In addition, the patient had no changes in medication and had maintained stable asthma control for several years, making drug-induced or seasonal influences less likely. Although we considered the exacerbation of asthma most suggestive of an effect of nemolizumab, we cannot fully exclude the contribution of other confounding factors, which represents another limitation of this report. The relationship between nemolizumab and asthma remains insufficiently understood; thus, further accumulation of cases and mechanistic studies will be essential to clarify this association and its underlying pathophysiology.

## Conclusions

PN is a chronic pruritic inflammatory disease driven by Th2-dominant immune dysregulation and neural hyperactivity. Conventional therapies often fail, while biologics such as dupilumab and nemolizumab have shown efficacy. However, nemolizumab has been reported to potentially induce cutaneous adverse events and exacerbate asthma, and in our case, a causal relationship between the drug and these events appeared highly likely. Careful monitoring is therefore warranted in susceptible patients. To date, quantitative evaluation of Th2-driven inflammation in the context of nemolizumab-related adverse events has been scarcely performed. Further accumulation of clinical data and additional research will be essential to elucidate the underlying mechanisms and to establish appropriate management strategies.
